# Integrin αIIbβ3 outside-in signaling activates human platelets through serine 24 phosphorylation of Disabled-2

**DOI:** 10.1186/s13578-021-00532-5

**Published:** 2021-02-08

**Authors:** Hui-Ju Tsai, Ju-Chien Cheng, Man-Leng Kao, Hung-Pin Chiu, Yi-Hsuan Chiang, Ding-Ping Chen, Kun-Ming Rau, Hsiang-Ruei Liao, Ching-Ping Tseng

**Affiliations:** 1 Department of Medical Biotechnology and Laboratory Science, College of Medicine, Chang Gung University, Taoyuan, 333 Taiwan, Republic of China; 2 Department of Medical Laboratory Science and Biotechnology, China Medical University, Taichung, 404 Taiwan, Republic of China; 3 Department of Laboratory Medicine, Chang Gung Memorial Hospital, Taoyuan, 333 Taiwan, Republic of China; 4 Department of Hematology-Oncology, E-Da Cancer Hospital, Kaohsiung, 824 Taiwan, Republic of China; 5 School of Medicine, College of Medicine, I-Shou University, Kaohsiung, 824 Taiwan, Republic of China; 6 Graduate institute of Natural Products, College of Medicine, Chang-Gung University, Taoyuan, 333 Taiwan, Republic of China; 7 Graduate institute of Biomedical Sciences, College of Medicine, Chang Gung University, Taoyuan, 333 Taiwan, Republic of China; 8 Department of Anesthesiology, Chang Gung Memorial Hospital, Taoyuan, 333 Taiwan, Republic of China; 9 Molecular Medicine Research Center, Chang Gung University, Taoyuan, 333 Taiwan, Republic of China

**Keywords:** Disabled-2, Phosphorylation, Platelet activation, Outside-in signaling

## Abstract

**Background:**

Bidirectional integrin αIIbβ3 signaling is essential for platelet activation. The platelet adaptor protein Disabled-2 (Dab2) is a key regulator of integrin signaling and is phosphorylated at serine 24 in eukaryotic cells. However, the mechanistic insight and function of Dab2-serine 24 phosphorylation (Dab2-pSer24) in platelet biology are barely understood. This study aimed to define whether and how Dab2 is phosphorylated at Ser24 during platelet activation and to investigate the effect of Dab2-pSer24 on platelet function.

**Results:**

An antibody with confirmed specificity for Dab2-pSer24 was generated. By using this antibody as a tool, we showed that protein kinase C (PKC)-mediated Dab2-pSer24 was a conservative signaling event when human platelets were activated by the platelet agonists such as thrombin, collagen, ADP, 12-O-tetradecanoylphorbol-13-acetate, and the thromboxane A2 activator U46619. The agonists-stimulated Dab2-pSer24 was attenuated by pretreatment of platelets with the RGDS peptide which inhibits integrin outside-in signaling by competitive binding of integrin αIIb with fibrinogen. Direct activation of platelet integrin outside-in signaling by combined treatment of platelets with manganese dichloride and fibrinogen or by spreading of platelets on fibrinogen also resulted in Dab2-pSer24. These findings implicate that Dab2-pSer24 was associated with the outside-in signaling of integrin. Further analysis revealed that Dab2-pSer24 was downstream of Src-PKC-axis and phospholipase D1 underlying the integrin αIIbβ3 outside-in signaling. A membrane penetrating peptide R11-Ser24 which contained 11 repeats of arginine linked to the Dab2-Ser24 phosphorylation site and its flanking sequences (RRRRRRRRRRR^19^APKAPSKKEKK^29^) and the R11-S24A peptide with Ser24Ala mutation were designed to elucidate the functions of Dab2-pSer24. R11-Ser24 but not R11-S24A inhibited agonists-stimulated Dab2-pSer24 and consequently suppressed platelet spreading on fibrinogen, with no effect on platelet aggregation and fibrinogen binding. Notably, Ser24 and the previously reported Ser723 phosphorylation (Dab2-pSer723) occurred exclusively in a single Dab2 molecule and resulted in distinctive subcellular distribution and function of Dab2. Dab2-pSer723 was mainly distributed in the cytosol of activated platelets and associated with integrin inside-out signaling, while Dab2-pSer24 was mainly distributed in the membrane fraction of activated platelets and associated with integrin outside-in signaling.

**Conclusions:**

These findings demonstrate for the first time that Dab2-pSer24 is conservative in integrin αIIbβ3 outside-in signaling during platelet activation and plays a novel role in the control of cytoskeleton reorganization and platelet spreading on fibrinogen.

## Background

Platelet activation is crucial for stopping blood loss following vessel wall damage and it plays a pivotal role in the onset of myocardial infarction and other thrombotic diseases. Bidirectional signaling of the platelet transmembrane receptor integrin αIIbβ3 is essential in the course of platelet activation [1]. Exposure of the circulating platelets to collagen and von Willebrand factor derived from the damaged endothelial cells turns on integrin inside-out signaling leading to an increase in the binding affinity of integrin αIIbβ3 to soluble fibrinogen [2]. Fibrinogen binding further activates integrin αIIbβ3 outside-in signaling that subsequently causes cytoskeletal remodeling and clot retraction to prevent blood loss [3–5].

Integrin αIIbβ3 bidirectional signaling is tightly regulated by different types of molecules including transmembrane proteins, protein kinases and adaptor proteins. Various signaling pathways are involved in the transmission of integrin αIIbβ3 inside-out signaling. The agonists of thrombin, thromboxane A2 (TXA2), and ADP bind to their respective G protein-coupled receptors and activate either a G_αq_-dependent increase in intracellular calcium and protein kinase C (PKC) activity, G_α12/13_-dependent Rho activation, G_αi_-dependent inhibition of adenylyl cyclase, and/or G_βγ_-dependent phosphoinositide 3-kinase-Akt activation [2, 3, 6–8]. Collagen interacts with glycoprotein VI and recruits Src and Syk to the plasma membrane followed by tyrosine phosphorylation of downstream substrates required for platelet activation [3, 9]. The phosphorylation and talin binding cause conformational change of the receptor and transform integrin from the resting to the active stage, which elicits high affinity binding activity to fibrinogen. The binding of fibrinogen further activates integrin outside-in signaling, causing the association of the cytoplasmic domain of integrin αIIbβ3 with actin filaments, intracellular signaling molecules, and adaptor proteins including talin, vinculin, zyxin, paxillin, filamin, α-actinin, and nonmuscle myosin IIA and IIB [10–13]. Therapeutic agents directly targeting αIIbβ3 are clinically valuable in the treatment of thrombotic diseases [14–16]. However, the αIIbβ3 blockers may still cause conformational changes of integrin and activate outside-in signaling with a risk of hemorrhagic complications [5, 17]. A better understanding of the components and mechanism of αIIbβ3 bidirectional signaling, in particular outside-in signaling, may provide novel and safer therapeutic targets without affecting normal hemostasis.

Disabled-2 (Dab2) is an adaptor protein expressed abundantly in human platelets [18–21]. Dab2 is distributed in the cytosol and α-granules in human platelets [19]. It is released from the α-granules and binds to either integrin αIIb or phospholipid sulfatide in response to platelet activation, thereby playing a role in platelet-fibrinogen and platelet-leukocyte adhesion and aggregation [19, 22–24]. Dab2 regulates inside-out signaling of integrin αIIbβ3 by playing a selective role in thrombin-stimulated G_α12/13_-mediated RhoA-ROCK activation in mouse platelets [21]. Thrombin also induces Dab2 phosphorylation at Ser723 for transmitting integrin inside-out signaling and causing Dab2-Cbl-interacting protein of 85 kDa (CIN85) complex disassociation during human platelet activation [20].

In eukaryotic cells, additional Dab2 phosphorylation sites have been identified by mass spectrometry and phosphoamino acid analysis [18, 25–34]. PKC-mediated Dab2-Ser24 phosphorylation inhibits 12-O-tetradecanoylphorbol-13-acetate (TPA)-induced AP-1 activity [28] and is involved in TPA-induced megakaryocytic differentiation of human leukemic K562 cells for maintaining integrin αIIbβ3 in an inactivated state [18]. Dab2-Ser249 phosphorylation is detected in resting and activated platelets induced by ADP but not thrombin [30]. A cluster of phosphorylation sites at residues 221–231, 324–329, and 393–401 are associated with either cell cycle arrest in mitosis or nuclear localization of Dab2 [27, 31–34]. However, the lack of phospho-Dab2 specific antibodies targeting the phosphorylation sites hampers the comprehensive analysis of these phosphorylation events in platelets and other cellular systems.

The Dab2-Ser24 phosphorylation site is highly conserved among different species. A phospho-Ser24-specific anti-Dab2 antibody was generated for elucidating the status and function of Dab2-Ser24 phosphorylation in human platelets. We defined in this study the Src-PKC signaling axis- and activated phospholipase D1 (PLD1)-mediated Dab2-Ser24 phosphorylation is a common event in agonist-stimulated human platelets and plays a pivotal function in integrin αIIbβ3 outside-in signaling.

## Results

### Generation and characterization of the phospho-Ser24-specific anti-Dab2 antibody

**Fig. 1:**
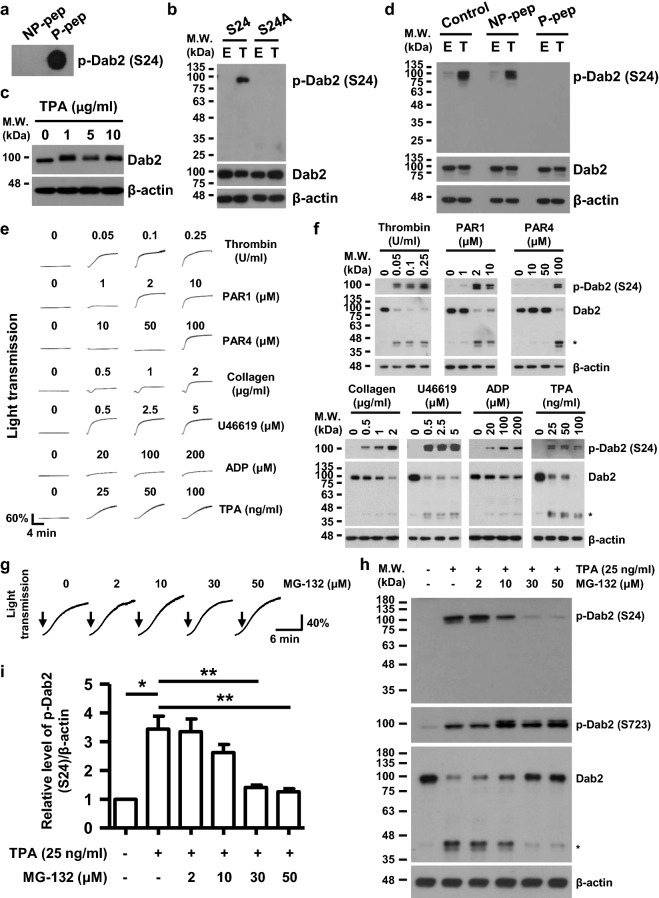
Ser24 phosphorylation and proteolytic cleavage of Dab2 during agonist-induced human platelet activation. **a** The Dab2 Ser24 phospho-peptide (P-pep) but not the non-phospho-peptide (NP-pep) was recognized by a dot blot assay using the anti-p-Dab2 (S24) antibody. **b** 293T cells were transfected with HA-Dab2 (S24) or HA-Dab2-S24A (S24A) plasmids then stimulated with ethanol (E) or TPA (T, 1 μg/ml) for 30 min. Lysates of 293T cells were analyzed by Western blotting using the indicated antibodies. **c**, **d** Human washed platelets were treated with the indicated concentrations of TPA under the thermomixer assay condition. Lysates of human platelets were analyzed by Western blotting using the indicated antibodies (panel c) or the anti-p-Dab2 (S24) antibody which has been pre-incubated with H_2_O (Control), P-pep or NP-pep as described in the peptide competition assays (panel d). **e**, **f** Human washed platelets were stimulated with the indicated concentrations of agonists and platelet aggregation was recorded by a platelet aggregometer (Chrono-Log). After 10 min, human washed platelets were lysed and the platelet lysates were collected for Western blotting using the indicated antibodies. The proteolytic cleavage products of Dab2 were marked as *. **g**, **h** Human washed platelets were pretreated with the indicated concentrations of MG-132 at RT for 30 min and then stimulated with TPA (25 ng/ml) and platelet aggregation was recorded by a platelet aggregometer (Chrono-Log). After 10 min, human washed platelets were lysed and the platelet lysates were collected for Western blotting using the indicated antibodies. Arrows indicate the addition of TPA (panel g). The proteolytic cleavage products of Dab2 were marked as * (panel h). **i** The level of Dab2 Ser24 phosphorylation was quantified by ImageJ software and normalized by the expression of β-actin. The level of Dab2 Ser24 phosphorylation in the resting platelet lysate was arbitrarily set as 1. The data are presented as the mean ± SEM of 3–4 independent experiments. *p < 0.05; **p < 0.01

Dab2-Ser24 and its flanking sequences are conserved among different species (Table 1). A phospho-Ser24-specific anti-Dab2 (anti-p-Dab2 (S24)) antibody was generated to facilitate the detection and analysis of Dab2-Ser24 phosphorylation in human platelets. Dot blot analysis revealed that the purified anti-p-Dab2 (S24) antibody recognized the phosphorylated peptide (P-pep) but not the non-phosphorylated peptide (NP-pep) (Fig. 1a).

**Table 1:** Dab2-Ser24 and its flanking sequences are conserved among different species

Species	AA#	P-Site	Sequence	GenBank accession number
Human	770	S24	^18^AAPKAPSKKEKKK^30^	NP_001334.2
Chimpanzee	770	S24	^18^AAPKAPSKKEKKK^30^	JAA20332.1
Rhesus Macaque	770	S24	^18^AAPKAPSKKEKKK^30^	AFI34301.1
Dog	770	S24	^18^AAPKAPSKKEKKK^30^	XP_536493.2
Platypus	758	S24	^18^TAPKVPSKKEKKK^30^	XP_001507342.2
Rat	768	S24	^18^AAPKAPSKKEKKK^30^	NP_077073.1
Mouse	766	S24	^18^AAPKAPSKKEKKK^30^	NP_075607.2
Hamster	765	S24	^18^AAPKVPSKKEKKK^30^	XP_003503146.1
Chicken	715	S26	^20^PPKAQTSKKEKKK^32^	XP_015133025.1
Frog	555	S24	^18^PTAKPPSKKEKKK^30^	NP_001165652.1
Zebra Danio	668	S21	^15^PPFKTPSKKEKKK^27^	XP_001920879.3

*AA#* number of amino acids, *P-Site* the amino acid corresponding to human Dab2 Ser24 phosphorylation site

Dab2-Ser24 is phosphorylated in eukaryotic cells treated with TPA [28]. The specificity of the anti-p-Dab2 (S24) antibody was confirmed by analysis of the lysates from TPA-treated 293T cells expressing the HA-Dab2 or the HA-Dab2-S24A plasmid, which encodes the HA-tag wild type and the serine to alanine mutation at the 24th amino acid residue of Dab2, respectively. The anti-p-Dab2 (S24) antibody was able to detect a band corresponding to Dab2 Ser24 phosphorylation in lysates from cells expressing HA-Dab2, but not HA-Dab2-S24A (Fig. 1b).

TPA treatment of human platelets under constant mixing in a thermomixer resulted in the retardation of Dab2 mobility when analyzed by sodium dodecyl sulfate–polyacrylamide gel electrophoresis (SDS-PAGE)(Fig. 1c). Western blotting using the anti-p-Dab2 (S24) antibody revealed that TPA induced Dab2-Ser24 phosphorylation in human platelets. The intensity of the protein band corresponding to Dab2-Ser24 phosphorylation was diminished when the antibody was pre-incubated with the P-pep but not the NP-pep peptide (Fig. 1d). These data indicate that an antibody that specifically recognizes Ser24 phosphorylation of Dab2 was generated and that TPA induced Dab2 phosphorylation at Ser24 in human platelets.

### Dab2-Ser24 phosphorylation is a conservative signaling during platelet activation

To elucidate whether or not Dab2-Ser24 phosphorylation is a common signaling event during platelet activation, human platelets were treated with different platelet agonists including thrombin, PAR1 peptide, PAR4 peptide, collagen, U46619, ADP or TPA under constant stirring in an aggregometer, which elicited greater platelet activation compared to the reaction under constant mixing in a thermomixer (data not shown). All agonists caused a dose-dependent increase in platelet aggregation (Fig. 1e). Dab2-Ser24 phosphorylation was induced when human platelets were treated with different platelet agonists. Dab2 underwent degradation and resulted in degradation products with the molecular weights of 38–45 kDa (Fig. 1f). TPA-stimulated platelets were used for elucidating whether or not Dab2 degradation is associated with Ser24 phosphorylation. The proteasome inhibitor MG-132 at high concentrations (30 and 50 μM) suppressed Dab2-Ser24 phosphorylation and degradation but not Dab2-Ser723 phosphorylation and platelet aggregation (Fig. 1g–i). These data indicate that Dab2-Ser24 phosphorylation is a common event during platelet activation and is associated with the degradation of Dab2 but not with platelet aggregation.

### Dab2-Ser24 is phosphorylated by PKCs during platelet activation

**Fig. 2:**
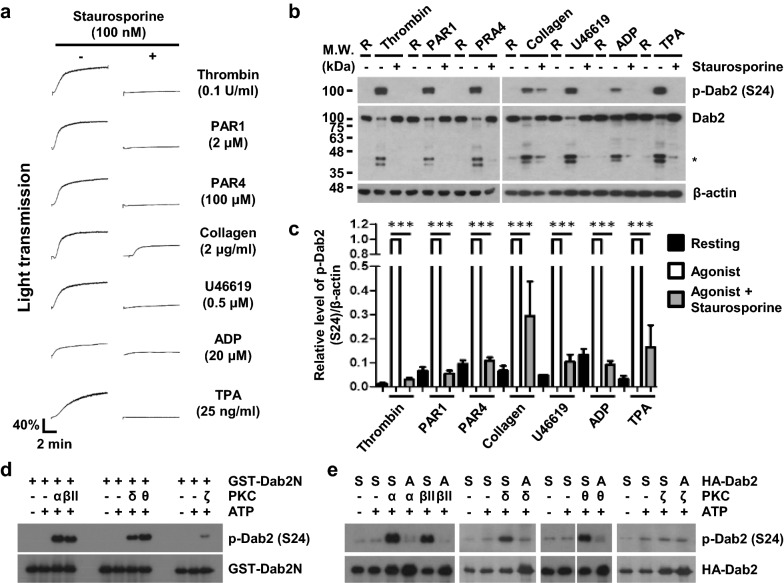
PKC isoforms are responsible for the phosphorylation of Dab2-Ser24. **a-c** Human washed platelets were pretreated with staurosporine (100 nM) for 5 min and then stimulated with the indicated concentrations of the agonists. Platelet aggregation was recorded by using a platelet aggregometer (Chrono-Log) (panel a). After 10 min, human washed platelets were lysed and the platelet lysates were collected for Western blotting using the anti-Dab2 (p96) and anti-p-Dab2 (S24) antibodies. The proteolytic cleavage products of Dab2 were marked as *. The expression of β-actin was used as a control of equal protein loading. R, resting platelets (panel b). The level of Dab2-Ser24 phosphorylation was quantified by ImageJ software and normalized by the expression of β-actin. The level of Dab2-Ser24 phosphorylation in agonist-stimulated platelet lysates was arbitrarily set as 1. The data are presented as the mean ± SEM of 4 independent experiments. ***p < 0.001 (panel c). **d** GST-Dab2N (1 ~ 234 amino acids) recombinant protein was used as the substrate for the in vitro protein kinase assay using the indicated PKC isoforms. The level of Dab2-Ser24 phosphorylation was analyzed by Western blotting using the anti-p-Dab2 (S24) antibody. The expression of GST-Dab2N recombinant protein was used for the control of equal protein loading. **e** HA-Dab2 (S) protein or HA-Dab2-S24A (A) protein from the lysates of 293T cells transfected with HA-Dab2 or HA-Dab2-S24A expression plasmids was enriched by immunoprecipitation using the anti-HA antibody. The immunoprecipitated proteins were used as the substrates for the in vitro protein kinase assay using the indicated PKC isoforms. The level of Dab2-Ser24 phosphorylation was determined by Western blotting using the anti-p-Dab2 (S24) antibody. The expression of HA-Dab2 or HA-Dab2-S24A was used for the control of equal protein loading

A peptide containing Ser24 and its flanking sequences (APS^24^KKEKKKGSEKTD) is a PKC substrate [28]. Platelets pre-treated with the pan-PKC inhibitor staurosporine, attenuated agonist-stimulated platelet aggregation (Fig. 2a) and inhibited Dab2-Ser24 phosphorylation (p < 0.001) and degradation (Fig. 2b, 2c). Human platelets express PKCα, βII, δ, θ, and ζ [35]. A GST-recombinant protein corresponding to the N-terminus of Dab2 (GST-Dab2N, amino acid 1–234) was phosphorylated by all PKC isoforms as seen with the in vitro protein kinase assay with PKCζ being the least effective (Fig. 2d). Ser24 in the full-length wild type HA-tag Dab2 recombinant protein (HA-Dab2-S24) was phosphorylated by PKCα, βII, δ and θ, but not PKCζ as seen with the in vitro immunocomplex protein kinase assay (Fig. 2e). This phosphorylation was abolished when Ser24 was mutated to Ala24 (HA-Dab2-S24A). These data support the perception that PKCα, βII, δ and θ are responsible for Ser24 phosphorylation of Dab2.

### Distinct phosphorylation of Dab2-Ser24 and -Ser723 in platelet activation

**Fig. 3:**
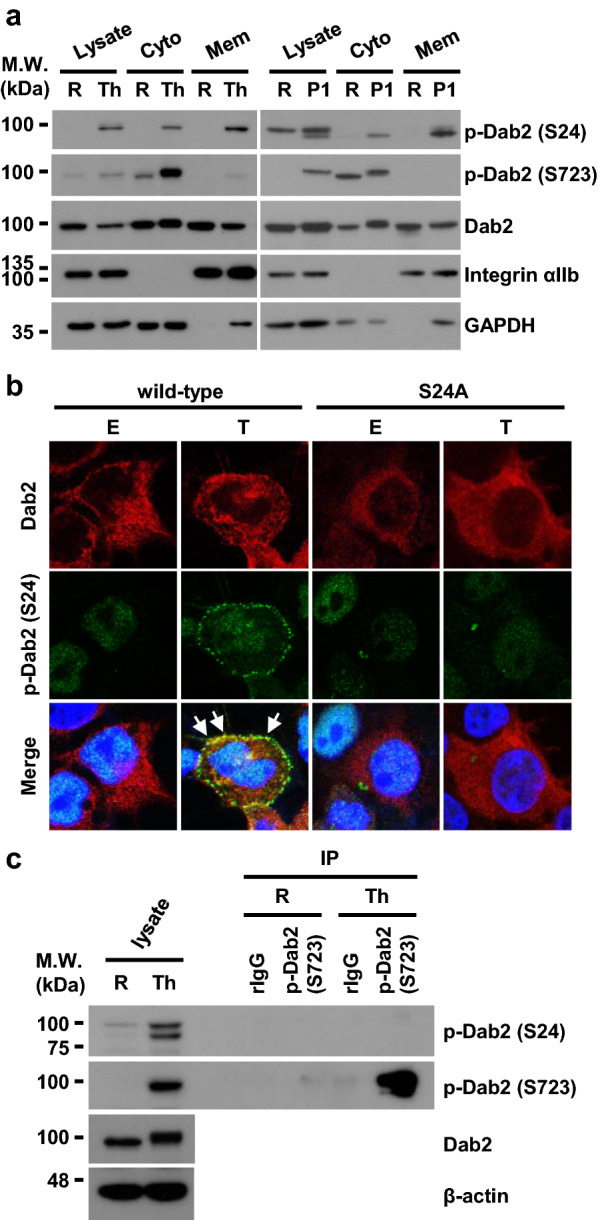
Ser24-phosphorylated Dab2 is mainly distributed in the membrane fraction of platelets. **a** Cytosolic (Cyto) and membrane (Mem) proteins were isolated from the resting (R) and thrombin (Th, 1 U/ml)- (left panel) or PAR1 peptide (P1, 10 μM)- (right panel) stimulated human platelet lysates using the Mem-PER Plus Membrane Protein Extraction Kit protocol. The proteins were subject to Western blotting using the indicated antibodies. GAPDH and integrin αIIb was used as the marker for the cytosolic and membrane fractions, respectively. Representative data of 2–3 independent experiments are shown. **b** 293T cells were transfected with HA-Dab2 (wild-type) or HA-Dab2-S24A (S24A) expression plasmids and then stimulated with ethanol (E) or TPA (T, 1 μg/ml) for 30 min. Immunofluorescence staining was then performed using the mouse anti-Dab2 (red) and the rabbit anti-p-Dab2 (S24) (green) primary antibodies followed by the Alexa Fluor 546-conjugated anti-mouse IgG and the Alexa Fluor 488-conjugated anti-rabbit IgG secondary antibody. Nucleated cells were defined by positive fluorescent staining of Hoechst 33342 (blue). Arrows indicate the co-localization of Dab2 and p-Dab2 (S24). **c** The lysates (2 mg) from the resting (R) or thrombin-stimulated (Th, 1 U/ml) human platelets were immunoprecipitated by the control rabbit IgG (rIgG) or anti-p-Dab2 (S723) antibody. The immunoprecipitated proteins (IP) were analyzed by Western blotting using the indicated antibodies. Representative data of 3 independent experiments are shown

Subcellular fractionation analysis of resting and thrombin- or PAR1 peptide-stimulated platelets revealed that Dab2 was present in the cytosolic and membrane fractions of resting and agonist-stimulated human platelets (Fig. 3a). Integrin αIIb and GAPDH were used as a marker of the membrane and cytosolic fractions of platelet lysates, respectively [36, 37]. Ser24-phosphorylated Dab2 was increased in thrombin- and PAR1 peptide-stimulated platelets and was mainly found in the membrane fraction, while Ser723-phosphorylated Dab2 was mainly found in the cytosolic fraction (Fig. 3a). 293T cells were transfected with either HA-Dab2-S24 or HA-Dab2-S24A expression plasmids. Ser24-phosphorylated Dab2 was mainly found in the cellular membrane fraction as shown by immunofluorescene staining analysis (Fig. 3b). These data indicate that Ser24-phosphorylated Dab2 is primarily found in the membrane fraction.

Thrombin-stimulated human platelets were subject to immunoprecipitation using the anti-pDab2 (Ser723) antibody for elucidating whether or not phosphorylation of Ser723 and Ser24 occurred in the same Dab2 molecule. Ser723- but not Ser24-phosphorylated Dab2 was found in the immunoprecipitated platelet lysates (Fig. 3c). These data indicate that Ser24 and Ser723 phosphorylations are mutually exclusive.

### Ser24-phosphorylated Dab2 underlies integrin outside-in signaling

**Fig. 4:**
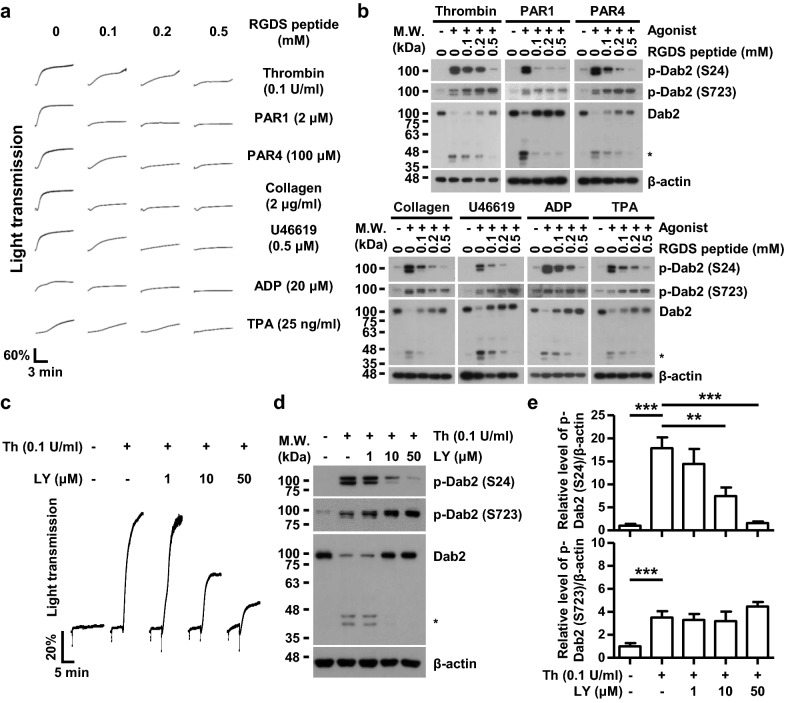
Blockage of integrin outside-in signaling attenuates Dab2-Ser24 phosphorylation stimulated by platelet agonists. **a**, **b** Human washed platelets were pre-incubated with the indicated concentrations of RGDS peptide at RT for 30 min and then stimulated with the indicated concentrations of the agonists. Platelet aggregation was recorded by a platelet aggregometer (Chrono-Log). After 10 min, human washed platelets were lysed and the platelet lysates were collected for Western blotting using the indicated antibodies. The proteolytic cleavage products of Dab2 were marked as *. Representative data of 2 independent experiments are shown. **c**, **d** Human washed platelets were pre-incubated with the indicated concentrations of LY333531 (LY) for 5 min and then stimulated with thrombin (Th, 0.1 U/ml). Platelet aggregation was recorded by a platelet aggregometer (Chrono-Log). After 10 min, human washed platelets were lysed and the platelet lysates were collected for Western blotting using the indicated antibodies. The proteolytic cleavage products of Dab2 were marked as *. **e** The level of Dab2-Ser24 phosphorylation and Dab2-Ser723 phosphorylation was quantified by ImageJ software and normalized by the expression of β-actin. The level of Dab2 phosphorylation in the resting platelet lysate was arbitrarily set as 1. The data are presented as the mean ± SEM of 5 independent experiments. **p < 0.01; ***p < 0.001

RGD-containing peptides inhibit fibrinogen binding consequently suppress platelet aggregation and outside-in signaling [38]. Human platelets were treated with different concentrations of the RGDS peptide prior to treatment with different types of agonists for determining whether or not Dab2-Ser24 phosphorylation is associated with integrin αIIbβ3 inside-out or outside-in signaling. The RGDS peptide suppressed agonist-stimulated platelet aggregation **(**Fig. 4a), Dab2-Ser24 phosphorylation and protein degradation (Fig. 4b). Dab2-Ser723 phosphorylation which associates with integrin inside-out signaling [20] was unaffected (Fig. 4b). These data indicate that blocking of integrin outside-in signaling suppresses Dab2-Ser24 phosphorylation.

PKCβ is the protein kinase for Ser24 and is the PKC isoform that mainly mediates integrin outside-in signaling in platelets [39]. Human platelets were pre-treated with the PKCβ-specific inhibitor, LY333531, [40] followed by thrombin stimulation for demonstrating that Dab2-Ser24 phosphorylation is involved in integrin outside-in signaling. Pretreatment of human platelets with LY333531 suppressed thrombin-stimulated platelet aggregation (Fig. 4c), Dab2-Ser24 phosphorylation and protein degradation (Fig. 4d) in a dose-dependent manner. Significant inhibition of Ser24 phosphorylation was observed when platelets were treated with LY333531 at 10 μM (p < 0.01) and 50 μM (p < 0.001), respectively. Dab2-Ser723 phosphorylation was unaffected (Fig. 4d, 4e). These data indicate that blocking PKCβ affects Dab2 phosphorylation in Ser24 and presumably this affects integrin outside-in signaling.

**Fig. 5:**
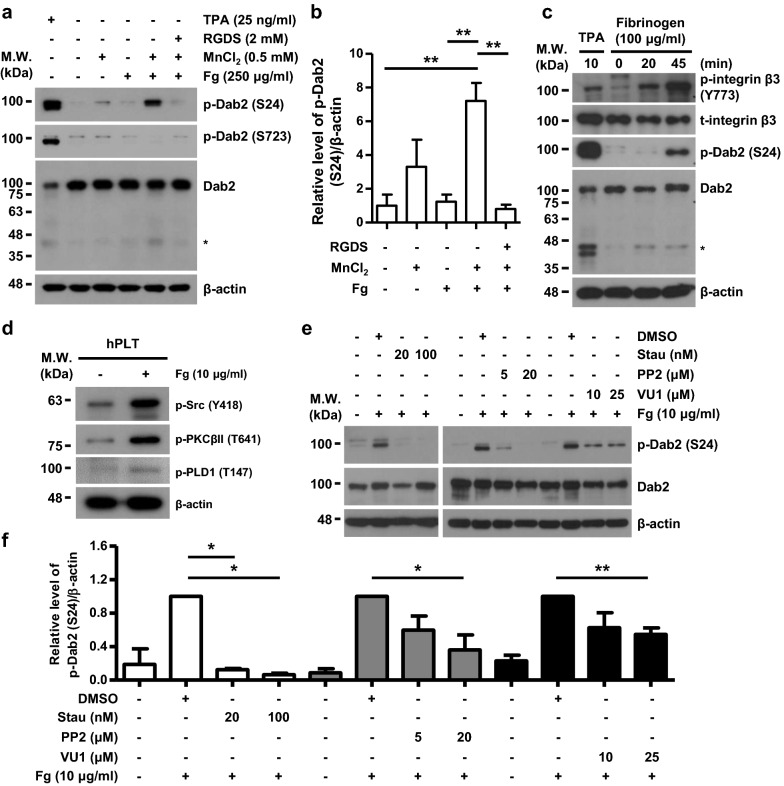
Integrin outside-in signaling induces Dab2-Ser24 phosphorylation. **a**, **b** Platelets with the indicated treatments were lysed for Western blotting analysis. The level of Dab2-Ser24 phosphorylation in the resting platelet lysate was arbitrarily set as 1. The data are presented as the mean ± SEM of 4 independent experiments. **c** Platelets with the indicated treatments were spread on 10-cm non-adhesive bacteriologic culture plates. The platelet lysates were collected for Western blotting analysis. Lysate from platelets treated with TPA (25 ng/ml) was used as a positive control. The proteolytic cleavage products of Dab2 were marked as *. **d** After spreading on coverslips, the platelet lysates were collected for Western blotting analysis. **e**, **f** Platelets were pre-incubated with the indicated concentrations of inhibitors then spread on coverslips. The platelet lysates were collected for Western blotting analysis. The proteolytic cleavage products of Dab2 were marked as *. The level of Dab2-Ser24 phosphorylation in platelets treated with DMSO and spread on Fg was arbitrarily set as 1. The data are presented as the mean ± SEM of 2–5 independent experiments. *p < 0.05; **p < 0.01

Manganese chloride (MnCl_2_) or fibrinogen alone did not result in a significant increase (p = 0.27 and p = 0.77, respectively) in Ser24 phosphorylation (Fig. 5a, 5b) comparing to no treatment group. Combined treatment of platelets with MnCl_2_ and fibrinogen increased Dab2-Ser24 but not -Ser723 phosphorylation. The increase in Dab2-Ser24 phosphorylation was diminished when platelets were pre-incubated with the RGDS peptide (Fig. 5a, b). Outside-in signaling is activated directly by plating platelets on fibrinogen-coated slides. Spreading of human platelets on immobilized fibrinogen resulted in a time-dependent phosphorylation of integrin β3 Tyr773 (Fig. 5c), a marker for integrin αIIbβ3 activation by outside-in signaling [41]. Integrin β3 expression was not altered as revealed by the antibody (t-integrin β3) which recognized integrin β3 protein. Dab2-Ser24 phosphorylation was also observed in the human platelets spreading on immobilized fibrinogen (Fig. 5c). These data indicate that the activation of integrin outside-in signaling is responsible for Dab2 Ser24 phosphorylation.

### Dab2-Ser24 phosphorylation mediated by the Src-PKC signaling axis and PLD1 activation regulates platelet spreading on fibrinogen

Integrin αIIbβ3 outside-in signaling activates Src and PKC signaling causing granule secretion, TXA2 synthesis and platelet spreading [39, 42]. PLD1 is also involved in outside-in signaling of integrin αIIbβ3 by supporting platelet adhesion, spreading and plug formation, thus stabilizing thrombus formation [43]. Spreading of human platelets on immobilized fibrinogen activated outside-in signaling and caused Src, PKC and PLD1 activation (Fig. 5d). Human platelets were pre-treated with the indicated inhibitors to define the upstream signaling involved in the regulation of Dab2-Ser24 phosphorylation. Fibrinogen-induced Dab2 phosphorylation at Ser24 was inhibited by pre-treatment of the platelets with staurosporine (Stau, a PKC inhibitor), PP2 (a Src inhibitor) or VU0155069 (VU1, a PLD1 inhibitor) compared to the platelets pre-incubated with the vehicle control DMSO, indicating that the integrin αIIbβ3 outside-in signaling activated the Src-PKC signaling axis and PLD1 activation leading to Dab2-Ser24 phosphorylation (Fig. 5e, f).

**Fig. 6:**
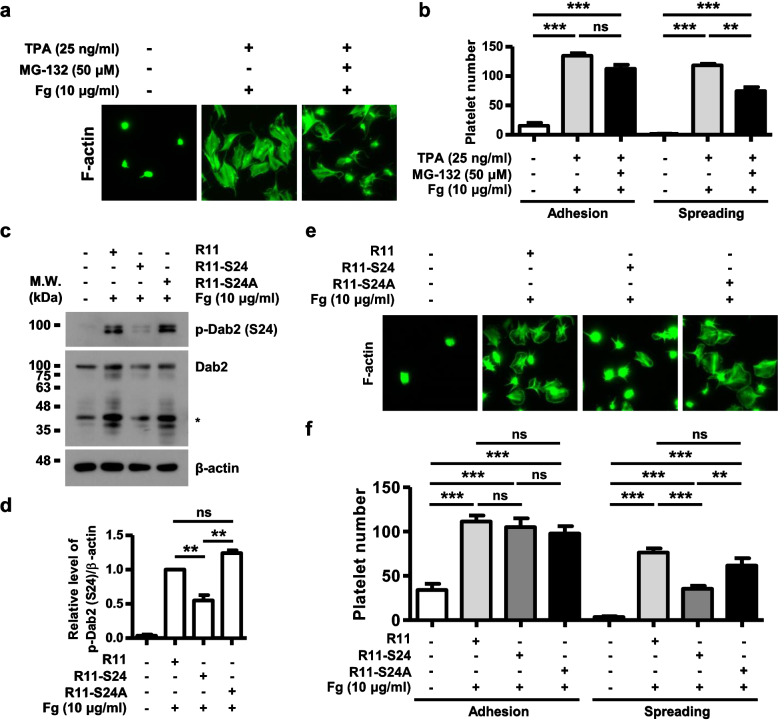
Dab2-Ser24 phosphorylation is involved in the regulation of cytoskeletal reorganization associated with outside-in signaling of integrin αIIbβ3. **a**, **b** Platelets with the indicated treatments were spread on coverslips. After spreading on coverslips, the platelets were fixed, permeabilized, stained for F-actin (green) and observed by phase contrast microscopy under a high power field (HPF) of 1,000 X magnification. The number of platelet adhesion/HPF and platelet spreading (area > 10 μm^2^)/HPF in each group is shown. The data are presented as the mean ± SEM of 2 independent experiments with four assays. ns, no significance. **c**, **d** After spreading on coverslips, the platelet lysates were collected for Western blotting analysis. The proteolytic cleavage products of Dab2 were marked as *. The level of Dab2-Ser24 phosphorylation in the platelets pretreated with R11 peptide was arbitrarily set as 1. The data are presented as the mean ± SEM of 2 ~ 6 independent experiments. **e**, **f** After spreading on coverslips, the platelets were fixed, permeabilized, stained for F-actin (green) and observed by phase contrast microscopy under a high power field (HPF) of 1,000 X magnification. The number of platelet adhesion/HPF and platelet spreading (area > 10 μm^2^)/HPF in each group is shown. The data are presented as the mean ± SEM of 2 ~ 4 independent experiments. ns, no significance. **p < 0.01; ***p < 0.001

Dab2-Ser24 phosphorylation was inhibited by MG-132 in TPA-stimulated human platelets (Fig. 1h, i). The effect of MG-132 on platelet adhesion and spreading on fibrinogen was further examined to determine the role of Dab2-Ser24 phosphorylation in integrin αIIbβ3 outside-in signaling. Pre-incubation of platelets with MG-132 (50 μM) had no effect on TPA-stimulated platelet adhesion but attenuated spreading on fibrinogen (p < 0.01) (Fig. 6a, b). The number of platelet adhesion per field was 15 ± 5, 135 ± 4 and 113 ± 7 for the mock-, TPA- and MG-132 plus TPA-treated group, respectively. The number of platelet spreading on fibrinogen per field was 2 ± 0, 118 ± 3 and 74 ± 7 for the mock-, TPA-, and MG-132 plus TPA-treated group, respectively.

Arginine polypeptide, a kind of cell-penetrating peptides, is used to study platelet functions [20, 44, 45]. R11 was linked to the Dab2 peptide Ser24 (R11-S24) sequences which contain the Dab2-Ser24 phosphorylation site and its flanking sequences. A peptide with R11 and Dab2 S24A mutation (R11-S24A) was also synthesized (Table 2). These peptides were delivered into platelets to explore the effects of these peptides on Dab2-Ser24 phosphorylation and platelet spreading on fibrinogen. Platelet Dab2-Ser24 phosphorylation and platelet adhesion and spreading on BSA were limited. Dab2-Ser24 was phosphorylated and platelets adhered and spread well on fibrinogen when platelets were pretreated with R11. R11-S24 but not R11-S24A peptide attenuated both Dab2-Ser24 phosphorylation and platelet spreading on fibrinogen without any effect on platelet adhesion (Fig. 6c–e). The number of platelet adhesion on fibrinogen per field was 112 ± 7, 105 ± 10 and 98 ± 8 for R11-, R11-S24- and R11-S24A-treated group, respectively. The number of platelet spreading on fibrinogen per field was 76 ± 5, 36 ± 6 and 62 ± 8 for the R11-, R11-S24- and R11-S24A-treated platelets, respectively (Fig. 6f). Consistent with these observations, pre-incubation of platelets with R11-S24 but not R11-S24A also attenuated thrombin-stimulated platelet spreading on fibrinogen when compared to the platelets pre-incubated with R11. No effect of R11-S24 was found on thrombin-stimulated platelet adhesion (Additional file 1: Fig. S1), fibrinogen binding, and platelet aggregation (Additional file 2: Fig. S2). These data indicate that Dab2-Ser24 phosphorylation is involved in the regulation of cytoskeletal reorganization associated with outside-in signaling of integrin αIIbβ3 but is not related to integrin αIIbβ3 inside-out signaling.

**Table 2:** List of peptides that used in this study

Peptide name	Amino acid sequences
*Antigen peptides*	
NP-pep	^20^PKAPSKKEKKKGP^32^C
P-pep	^20^PKAPpSKKEKKKGP^32^C
*Cell-penetrating peptides*	
R11	RRRRRRRRRRR
R11-S24	RRRRRRRRRRR^19^APKAPSKKEKK^29^
R11-S24A	RRRRRRRRRRR^19^APKAPAKKEKK^29^

*pS* phospho-serine

## Discussion

αIIbβ3 is the dominant integrin on platelet membranes and it is essential for normal platelet function, hemostasis and thrombosis [1]. Integrin αIIbβ3 signaling is a complex process that is tightly and delicately regulated by several types of proteins, such as the transmembrane proteins, adaptor molecules, kinases, phosphatases and the Rho-family small GTPases. In addition to its phosphorylation at Ser723 [20], Dab2 is phosphorylated at Ser24 by PKC when platelets are activated by agonists. Ser24-phosphorylated Dab2 is found mainly in the platelet membrane. It sustained integrin αIIbβ3 outside-in signaling and is associated with Dab2 protein degradation.

Considerable effort is still needed to fully explore how integrin αIIbβ3 interacts with its regulatory proteins, and how its regulatory proteins interact with one another in space and time. The PTB domain of Dab2 interacts with the non-phosphorylated NPXY motifs located at the amino acids 744–747 and 756–759 of the integrin β3 cytoplasmic tail [11, 18]. Dab2 when released from activated platelets interacts with the fibrinogen binding site of integrin αIIb and the phospholipid sulfatide on the outer surface of the platelet membrane [19, 22–24]. The balance of Dab2 between sulfatide- and integrin receptor-bound states is involved in the control of the extent of the clotting response [22]. The involvement of Dab2 in integrin αIIbβ3 signaling is complicated by the change in Dab2 phosphorylation during platelet activation. This and our previous study [20] defined Dab2 phosphorylation at Ser24 and Ser723 in human platelets, respectively. Although the phosphorylaton of these two amino acids occurred during agonist**-**stimulated platelet activation, they have unique characteristics and play distinct roles in integrin signaling. First, Ser723 and Ser24 phosphorylation of Dab2 was induced by integrin inside-out and outside-in signaling, respectively. This is supported by the finding that blockage of integrin outside-in signaling with the RGD peptide inhibited agonist-induced Ser24 but not Ser723 phosphorylation. Ser24 but not Ser723 phosphorylation was induced by plating platelets on fibrinogen or treating platelets with fibrinogen and MnCl_2_. Both approaches have been shown to directly initiate integrin outside-in signaling [39]. Second, Ser723- and Ser24-phosphorylated Dab2 have distinct functions in integrin inside-out and outside-in signaling. Ser723 phosphorylation of Dab2 underlies thrombin-stimulated inside-out signaling of integrin and, by regulating the interaction between Dab2 and CIN85, provides an alternative pathway in the control of ADP/dense granule release, fibrinogen binding, platelet aggregation, and integrin αIIbβ3 activation [20]. Ser24 phosphorylation of Dab2 was mediated by outside-in signaling of integrin and controlled platelet spreading on fibrinogen, as evidenced by the fact that the R11-S24 peptide inhibited Dab2 Ser24 phosphorylation and platelet spreading on fibrinogen. These studies thereby suggest that Dab2 plays a dual role in inside-out and outside-in signaling of integrin αIIbβ3 through multiple mechanisms including the Dab2-integrin αIIbβ3 interaction and phosphorylation at specific amino acid residues of Dab2. This study adds to the list of adaptor proteins, in addition to talin [5], that have a dual function in the regulation of inside-out and outside-in signaling of integrin αIIbβ3.

Several findings in the current study may explain how Dab2 elicits distinct functions in bidirectional integrin signaling. Dab2-Ser24 phosphorylation appears to correlate with the degree of platelet aggregation stimulated by the agonists. Attenuation of Dab2-Ser24 phosphorylation but not platelet aggregation by the proteasome inhibitor MG-132 indicates that Dab2-Ser24 phosphorylation is not related to integrin inside-out signaling and platelet aggregation. The mechanism for dephosphorylation of Dab2-Ser24 may attribute to the increase in phosphatase activity by MG-132. As described in previous studies, MG-132 promotes protein phosphatase 2A to dephosphorylate endothelial nitric oxide synthase that governs the nitric oxide-dependent signaling pathways in vascular endothelial cells [46]. It also activates dual specificity phosphatases to systemically perturb the intracellular phosphoproteome and the transition of kinase cascade signaling [47]. Hence, MG-132 may potentially regulate integrin signaling via its regulation of Dab2-Ser24 phosphorylation.

Dab2-Ser24 phosphorylation was not detected in the protein lysate immunoprecipitated by the anti-Ser723-Dab2 antibody, indicating that a single Dab2 protein is phosphorylated exclusively either at Ser24 or Ser723. Ser24- and Ser723-phosphorylated Dab2 was found mainly in the platelet membrane and the cytosolic fraction, respectively. There are at least two pools of Dab2 proteins within the agonist-stimulated platelets. Ser723-phosphorylated Dab2 found in the cytosolic fraction of platelets was involved in integrin inside-out signaling. Dab2-Ser723 phosphorylation causes the dissociation of Dab2-CIN85 complex and regulates αIIbβ3 activation and ADP release leading to an increase in fibrinogen binding and platelet aggregation in thrombin‐stimulated platelets [20]. Ser24-phosphorylated Dab2 found in the membrane fraction of platelet mediated integrin outside-in signaling. Mutually exclusive phosphorylation of specific amino acid residues has been employed by other proteins in generating specificity and pleiotropy in biological systems [48, 49]. The mechanisms for mutually exclusive phosphorylation of Ser24 and Ser723 are still not clear. Phosphorylation at either Ser24 or Ser723 may cause spatial hindrance that exclude the access of protein kinase to the other phosphorylation site. It is also likely that the spatial association of Dab2 and specific PKC isoform(s) within a specific compartment favors one of the phosphorylation sites over the other and determines the specificity of PKC isoforms in the phosphorylation of Dab2 at Ser24 or Ser723. Mutually exclusive phosphorylation of Dab2 may thereby play an important role in generating specificity and pleiotropy of integrin signaling.

The signaling axis leading to Dab2-Ser24 phosphorylation was investigated in this study. PKC isoforms in integrin signaling play a specific role in platelet activation [50]. PKCα regulates dense granule secretion and in parallel reduces collagen-related peptide-induced αIIbβ3 activation and platelet aggregation in response to submaximal agonist concentrations [51]. PKCβ is recruited to αIIbβ3 via RACK1 and is involved in the regulation of platelet spreading on fibrinogen or integrin activation by Mn^2+^ and soluble fibrinogen [39]. PKCδ plays an essential role in platelet signaling, integrin αIIbβ3 activation, and TXA2 release [52]. PKCθ is required for hemostasis and the positive regulation of thrombin-induced platelet aggregation and α-granule secretion [35, 53]. PKCη positively regulates agonist-induced thromboxane generation with no effect on platelet aggregation [54]. Although most PKC isoforms serve as the protein kinases for Dab2-Ser24 in the in vitro protein kinase assays, PKC isoforms usually elicit substrate specificity in vivo. Consistent with the role of PKCβ and Dab2-Ser24 phosphorylation in outside-in signaling of integrin, Ser24 phosphorylation of Dab2 in human platelets was inhibited by pretreatment of the platelets with a PKCβ inhibitor. Together with the inhibition of Ser24 phosphorylation by the Src inhibitor (PP2) and the PKC inhibitor (staurosporine), the signaling axis of integrin-Src-PKCβ-Dab2 may mediate Dab2-Ser24 phosphorylation associated with integrin outside-in signaling. On the other hand, Dab2-Ser24 phosphorylation wa**s** inhibited by pretreatment of platelets with the PLD1 inhibitor (VU0155069). PKC is downstream of PLD activation and signaling [55, 56] and PLD mainly functions in the cleavage of phospholipids to produce phosphatidic acid which metabolize to lysophosphatidic acid or to the PKC activator diacylglycerol [57]. Instead of direct regulation of Dab2-Ser24 phosphorylation, PLD most likely plays a role in activating PKC and provides a positive feedback loop to stimulate Dab2-Ser24 phosphorylation.

The molecular details regarding how Dab2-Ser24 phosphorylation regulates platelet spreading on fibrinogen and integrin outside-in signaling remains to be fully explored. Based on the data of this study, Dab2 may act as a negative regulator of outside-in signaling and form molecular complexes with integrin-associated proteins and/or phospholipids. Dab2-Ser24 phosphorylation may fine-tune the interaction between Dab2 and the integrin-associated signaling molecules, release its suppressive function in outside-in signaling and cause platelet spreading on fibrinogen. To assure full activation of outside-in signaling, Dab2 thereby undergoes degradation after phosphorylation. We noted that these findings are not in accord with our previous study using TPA-mediated megakaryocytic differentiation of K562 cells as the model system which revealed that Dab2-Ser24 phosphorylation acts as a negative regulator in αIIbβ3 inside-out signaling [18]. The different functions of Dab2-Ser24 phosphorylation in integrin signaling may be attributed to the use of different experimental approaches and model systems. The involvement of Dab2-Ser24 phosphorylation in integrin inside-out signaling is mainly based on ectopic expression of wild type and S24A-mutated Dab2 in the K562 cells. Wild type Dab2 but not the S24A mutant associates with membrane and interacts with integrin β3 leading to a decrease in αIIbβ3 activation and αIIbβ3-mediated fibrinogen adhesion (inside-out signaling) [18]. In order to transform K562 cells into megakaryocyte-like cells, most of these analyses were performed with long term treatment of TPA (48 h). In the current study, the functional role of Dab2 in outside-in signaling was directly addressed using platelet as the model system with short term treatment of agonists (10–45 min). With the different cellular content of the model systems, the integrin-related signaling proteins may have distinct components with various spatial and temporal regulation and assembly of protein complexes, and enzymatic activity (such as PKC) in platelets and the megakaryocytic differentiated K562 cells. Ser24-phosphorylated Dab2 may thereby interact with different subsets of integrin signaling proteins and elicit different functional roles in integrin signaling. Moreover, the controversial results may due to different combinations of phosphorylation of various additional sites in platelets and K562 cells. In this regard, Dab2 phosphorylation at the amino acid residues of Ser224, Ser249, Ser394, and Ser401 has been reported in agonist**-**stimulated platelet lysates [20, 30]. We could not rule out the contributions of other phosphorylation or molecular events that together with Dab2-Ser24 phosphorylation in integrin signaling. Generation of an antibody specific to the phosphorylated amino acid residues and further investigation of the underlying functions of these phosphorylation events are required.

In addition to providing new insights into the function of Dab2-Ser24 phosphorylation in integrin αIIbβ3 outside-in signaling, a Dab2-Ser24 specific antibody and a R11-Dab2 peptide with inhibitory activity on Ser24 phosphorylation proved useful for functional studies of Dab2-Ser24 phosphorylation in human platelets. Dab2 phosphorylation is involved in endocytosis, cell cycle progression, and cell proliferation [18, 25–34]. The Dab2-Ser24 phospho-specific antibody and the R11-Dab2 cell penetrating peptide are valuable tools for exploring the functional role of Dab2-Ser24 phosphorylation in other cellular processes.

## Conclusions

**Fig. 7:**
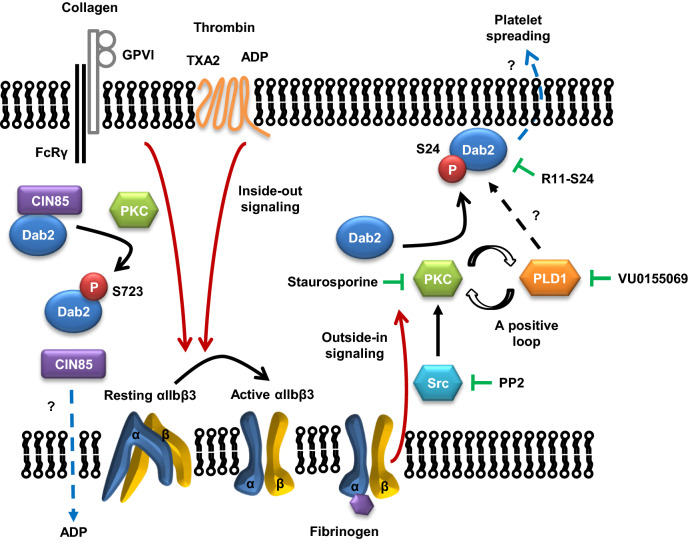
Proposed model for the dual role of Dab2 phosphorylations in platelet activation. Two pools of Dab2 are present in human platelets. PKC-mediated Dab2 Ser723 phosphorylation mainly distributed in the cytosolic fraction causes the dissociation of Dab2-CIN85 protein complex upon agonist-stimulated integrin inside-out signaling. The molecular consequences for the dissociation of Dab2-CIN85 protein complex is related to ADP release, integrin αIIbβ3 activation, fibrinogen binding and platelet aggregation. Activation of the Src-PKC signaling axis and PLD underlying integrin outside-in signaling causes Dab2-Ser24 phosphorylation, which is mainly distributed in the membrane fraction and subsequently regulates platelet spreading on fibrinogen. PP2 (a Src inhibitor), staurosporine (a PKC inhibitor), VU0155069 (a PLD1 inhibitor) and R11-S24 peptide suppress Dab2-Ser24 phosphorylation

Based on the findings of the current and previous studies [20, 58], a model for the function and regulation of Dab2 phosphorylation in platelet signaling is proposed (Fig. 7). Distinct pools of Ser723- and Ser24-phosphorylated Dab2 are present in agonist-stimulated human platelets. Ser723 phosphorylation induced by platelet agonists regulates Dab2-CIN85 protein complexes and transmits integrin inside-out signaling, leading to activation of integrin αIIbβ3 [20]. Novel evidence presented in the current study shows that Dab2-Ser24 phosphorylation induced by the integrin-Src-PKC and PLD1 signaling axis mediates integrin αIIbβ3 outside-in signaling and plays a pivotal role in platelet spreading on fibrinogen. This study sheds light on the molecular basis of integrin αIIbβ3 signaling in human platelets. Better understanding of integrin αIIbβ3 signaling could provide greater insight into the mechanism of thrombus formation, leading to the development of new therapeutic agents for counteracting clot formation.

## Methods

### Reagents

Thrombin, fibrinogen and ADP were purchased from Calbiochem (Darmstadt, Germany). The collagen was purchased from Chrono-Log (Havertown, PA). Apyrase, TPA, MnCl_2_, anti-HA agarose, Freund’s adjuvant complete and Freund’s adjuvant incomplete were purchased from Sigma (St Louis, MO). Prostaglandin I_2_ (PGI_2_) and U46619 were purchased from Cayman Chemical (Ann Arbor, MI). LY333531 was purchased from MedChemExpress (Monmouth Junction, NJ). The anti-Dab2 (p96) antibody was purchased from BD Biosciences (San Diego, CA). The anti-β-actin antibody was purchased from Novus Biological (Mill Valley, CA). The anti-p-integrin β3 (Y773) antibody and Lipofectamine 2000 reagent were purchased from invitrogen (Carlsbad, CA). The anti-t-integrin β3 (N20) antibody was purchased from Santa Cruz Biotechnologies (Santa Cruz, CA). The protein kinases were purchased from SignalChem (Richmond, BC). Acti-stain 488 phalloidin was purchased from Cytoskeleton inc. (Denver, CO). Amicon Ultra-15 Centrifugal Filter Units (3 K) was purchased from Merck Millipore (Darmstadt, Germany). PAR1 peptide (SFLLRN-NH2, purity > 95%), PAR4 peptide (AYPGKF-NH2, purity > 95%), R11 peptide (RRRRRRRRRRR, purity > 95%), R11-S24 peptide (RRRRRRRRRRR^19^APKAPSKKEKK^29^, purity > 95%), R11-S24A peptide (RRRRRRRRRRR^19^APKAPAKKEKK^29^, purity > 95%) and RGDS peptide were synthesized by Kelowna international Scientific inc. (Taiwan).

### Generation of the phospho-Ser24-specific anti-Dab2 antibody

The synthetic peptide (antigen) corresponding to the amino acid residues 20–32 of human Dab2 (Table 2) was synthesized and conjugated with keyhole limpet hemocyanin by 2% glutaraldehye. The peptide (1 mg/mL) was mixed with 1 ml Freund’s adjuvant complete and was subcutaneously injected into the rabbit. After a second immunization, the blood was collected and the antibody was purified by using a two-step affinity column purification protocol and concentrated by Amicon Ultra-15 Centrifugal Filter Units (3 K).

### Dot blot assay

The peptides (20 nmol) were spotted onto the nitrocellulose membrane. The membrane was air-dried and blocked with 5% non-fat milk in 0.05% TBS-T (20 mM Tris–HCl, 150 mM NaCl, pH 7.5 and 0.05% Tween-20) for 30 min followed by protein detection as described in Western blot analysis.

### Western blot analysis

Western blotting was performed as described previously [20, 59]. Proteins were separated by SDS-PAGE under reducing condition, transferred to a polyvinylidene fluoride membrane and blocked with 5% non-fat milk for 1 h. The membrane was incubated with primary antibody at room temperature (RT) for 1.5 h followed by secondary antibody for 1 h. Protein expression was detected by ECL-Plus reagents. The level of Dab2-Ser24 phosphorylation was quantified by ImageJ software and normalized by the expression of β-actin.

### Cell culture, transfection and immunoprecipitation

The 293T cells were cultured in Dulbecco's modified eagle medium supplemented with 5% fetal bovine serum and were transfected with plasmid DNA using the Lipofectamine 2000 reagent as described previously [60]. For immunoprecipitation, 2 mg of protein were incubated with 20 μl of anti-HA agarose or 2 μg of the indicated antibody at 4 °C for 2.5 h. The protein complexes were washed five times with 1X lysis buffer and readied for the in vitro protein kinase assay.

### Peptide competition assay

The peptide competition assay was performed using phosphorylated or non-phosphorylated peptides with a phospho-specific primary antibody, where an antibody concentration of 1 μg/ml and a 200-fold molar excess of peptide were used in a total reaction volume of 3 ml. Antibodies with peptides were incubated at room temperature for 30 min with gentle rocking. The immune complexes were removed by centrifugation at 4 °C and 13,200 rpm for 15 min. The supernatant was collected carefully. The pre-incubated antibodies in each sample were ready for use.

### Human washed platelets preparation

Whole blood from healthy donors was mixed with sodium citrate (3.15%) in the ratio of nine to one and was centrifuged at 200*g* for 20 min to obtain platelet-rich-plasma (PRP). Platelets were then obtained by centrifugation of PRP at 980*g* for 10 min in the presence of PGI_2_ (0.3 μg/ml). After washing twice with Tyrode’s buffer [61] supplemented with PGI_2_ (0.3 μg/ml) and apyrase (0.02 U/ml), the washed platelets were resuspended in Tyrode’s buffer.

### Platelet activation assay

In thermomixer assay condition, 200 μl of washed platelets (3 × 10^8^/ml) was added into an eppendorf tube and stimulated with the indicated agonists at 37 °C under stirring at 500 rpm for 10 min. For the aggregometer (Chrono-Log) assay, 500 μl of washed platelets (3 × 10^8^/ml) was added into a cuvette with a stirring bar and stimulated with the indicated agonists at 37 °C under stirring at 1000 rpm for 10 min. After the addition of 5× lysis buffer (50 mM Tris–HCl (pH 7.4), 500 mM NaCl, 2.5 mM CaCl_2_, 2.5 mM MgCl_2_, 5% Triton X-100, 50 μg/ml aprotinin, 50 μg/ml leupeptin, 5 mM phenylmethylsulfonyl fluoride (PMSF), 1 mM sodium orthovanadate, 50 mM sodium fluoride, and 5 mM EGTA), samples were collected for Western blotting.

### In vitro protein kinase assay

HA-Dab2 or HA-Dab2-S24A was immunoprecipitated by the use of anti-HA agarose from 293T cells overexpressing the indicated Dab2 proteins. In vitro protein kinase assays were performed by incubating the immunoprecipitated proteins or the GST-Dab2N recombinant protein with the kinase buffer [28] in the presence of the indicated protein kinases at 30 °C for 30 min. The assay was then terminated by adding 5× sample buffer.

### Subcellular fractionation of platelet lysates

Subcellular fractionation of platelet lysates was performed according to the manufacturer’s instruction of Mem-PER Plus Membrane Protein Extraction Kit (ThermoFisher). Platelets were obtained by centrifugation at 980*g* for 10 min and lysed by 750 μl Permeabilization Buffer at 4 °C rotated for 10 min. Cytosolic proteins were collected by centrifugation at 16,000*g* for 15 min. The pellet was incubated with 500 μl of Solubilization Buffer at 4 °C rotated for 30 min. Membrane-associated proteins were collected by centrifugation at 16,000*g* for 15 min.

### Immunofluorescence staining and F-actin staining

Cells or adhered platelets were fixed with 4% paraformaldehyde at RT for 10 min and then washed twice with 1× PBS. Fixed cells were permeabilized with 1× PBS containing 0.1% Triton X-100 at RT for 10 min and then washed twice with 1× PBS. For immunofluorescence staining, cells were blocked with 3% bovine serum albumin (BSA) at RT for 30 min and then incubated with the mouse anti-Dab2 (p96) or the rabbit anti-p-Dab2 (S24) antibody at RT for 60 min. After washing twice with 1× PBS, cells were incubated with the Alexa Fluor 546-conjugated anti-mouse IgG secondary antibody or Alexa Fluor 488-conjugated anti-rabbit IgG secondary antibody at RT for 40 min. Nucleated cells were defined by positive fluorescent staining of Hoechst 33,342. For F-actin staining, platelets were stained with acti-stain 488 phalloidin (100 nM) at RT for 30 min and then washed twice with 1× PBS. Stained cells on coverslips were mounted with Dako mounting medium and observed by microscopy.

### Fibrinogen‐induced outside‐in signaling assay

The 10-cm non-adhesive bacteriologic culture plates were pre-coated with 100 μg/mL fibrinogen and kept at 4 °C overnight. After blocking with heat-denatured (70 °C for 1 h) 5% BSA, 2 ml of washed platelets (3 × 10^8^/ml) was added and incubated at 37 °C for the indicated period of time. For inhibitors and the R11 peptide (Table 2) assay, 24 × 60 mm coverslips were pre-coated with 10 μg/mL fibrinogen and kept overnight at 4 °C. After blocking with heat-denatured 5% BSA, 2 ml of the washed platelet (3 × 10^8^/ml) was pre-treated with R11, R11-Dab2 peptide (375 nM) or the indicated concentrations of the inhibitors at RT for 5 min. The platelets were then added onto the coverslips and incubated at 37 °C for 45 min. Platelet adhesion and spreading on BSA-blocked coverslips was used as a control group in this assay. After rinsing twice with Tyrode’s buffer, the reaction was stopped, and the lysates of the platelets adherent to fibrinogen were prepared by adding 1× lysis buffer.

### Fibrinogen binding assay

Platelets were stimulated with indicated agonists and incubated with Alexa Fluor 488-conjugated fibrinogen (10 μg/ml) for 20 min at room temperature in the dark. The reactions were stop by adding 400 μl 1× PBS and the samples were analyzed within 30 min using the Accuri C6 Flow Cytometer with CFlow® Software (BD Biosciences).

### Statistical analysis

Student’s t test was used for all analyses. The data were presented as the mean ± standard error of the mean (SEM). p < 0.05 was considered statistically significant.

## Supplementary Information

**Additional file 1: Figure S1.** Pre-incubation of platelets with R11-S24 suppresses platelet spreading on fibrinogen during thrombin stimulation. Platelets were pre-incubated with R11, R11-S24 or R11-S24A peptide then stimulated with thrombin (0.05 U/ml) and were spread on coverslips. After spreading on coverslips, the platelets were fixed, permeabilized, stained for F-actin (green) and observed by phase contrast microscopy.

**Additional file 2: Figure S2. **Pre-incubation of platelets with R11-S24 had no effect on thrombin-induced fibrinogen binding and platelet aggregation. Platelets were pre-incubated with R11, R11-S24 or R11-S24A peptide then stimulated with thrombin (0.05 U/ml). Fibrinogen binding was determined by flow cytometry. Platelet aggregation was recorded by a platelet aggregometer.

## Data Availability

All data in this study are available upon request.

## Abbreviations

BSA: Bovine serum albumin
CIN85: Cbl-interacting protein of 85 kDa
Dab2: Disabled-2
Dab2-pSer24: Dab2-Ser24 phosphorylation
NP-pep: Non-phosphorylated peptide
P-pep: Phosphorylated peptide
PGI_2_: Prostaglandin I_2_
PKC: Protein kinase C
PLD1: Phospholipase D1
PMSF: Phenylmethylsulfonyl fluoride
PRP: Platelet-rich-plasma
RT: Room temperature
SDS-PAGE: Sodium dodecyl sulfate–polyacrylamide gel electrophoresis
SEM: Standard error of the mean
TPA: 12-O-tetradecanoylphorbol-13-acetate
TXA2: Thromboxane A2
