# Supplementary material for: Integrin αIIbβ3 outside-in signaling activates human platelets through serine 24 phosphorylation of Disabled-2
Source: Cell Biosci. 2021 Feb 8;11:32. doi: 10.1186/s13578-021-00532-5 (PMC7869483; doi:10.1186/s13578-021-00532-5)
Supplement: Supplementary file 1 — Additional file 1: Figure S1. Pre-incubation of platelets with R11-S24 suppresses platelet spreading on fibrinogen during thrombin stimulation. Platelets were pre-incubated with R11, R11-S24 or R11-S24A peptide then stimulated with thrombin (0.05 U/ml) and were spread on coverslips. After spreading on coverslips, the platelets were fixed, permeabilized, stained for F-actin (green) and observed by phase contrast microscopy. [file 13578_2021_532_MOESM1_ESM.pdf]

Fig. S1

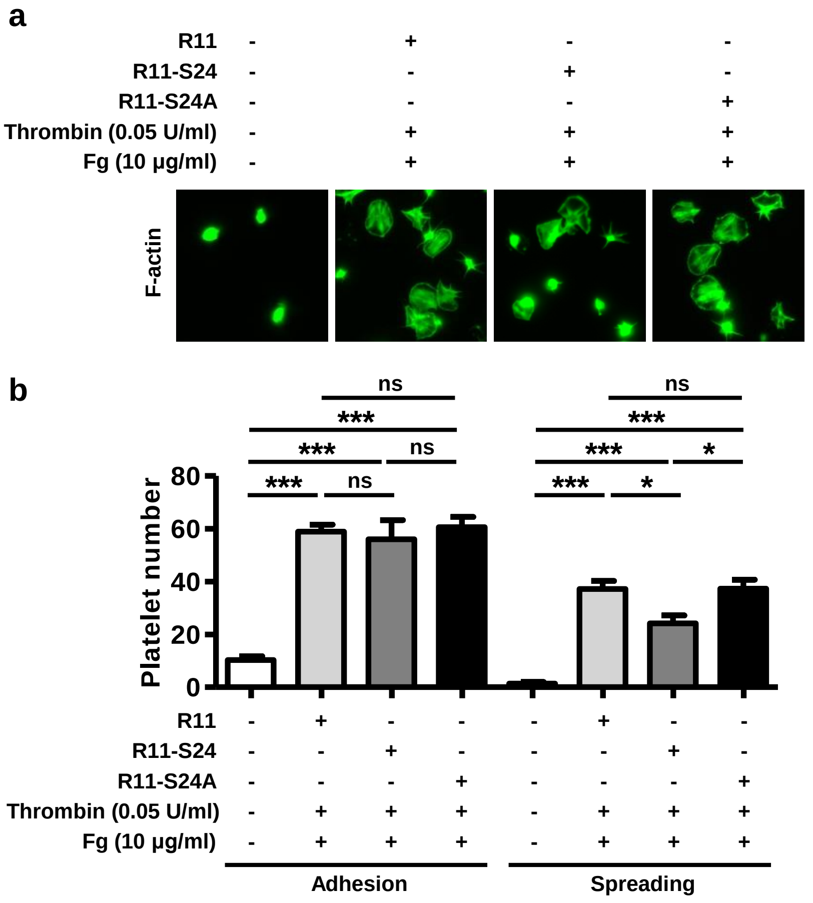

Fig. S1 Pre-incubation of platelets with R11-S24 suppresses thrombin-stimulated platelet spreading on fibrinogen. **a-b** Platelets with the indicated treatment were spread on coverslips. After spreading on coverslips, the platelets were fixed, permeabilized, stained for F-actin (green) and observed by phase contrast microscopy under a high power field (HPF) of 1,000 X magnification. The number of platelet adhesion and platelet spreading (area > 10 μm^2) was counted in 3 HPFs per group. The number of platelet adhesion/HPF and platelet spreading/HPF in each group are shown. The data are presented as the mean ± SEM of 2 independent experiments with analysis for a total of 6 HPFs. ns, no significance. *p < 0.05; **p < 0.01
