# Supplementary material for: Integrin αIIbβ3 outside-in signaling activates human platelets through serine 24 phosphorylation of Disabled-2
Source: Cell Biosci. 2021 Feb 8;11:32. doi: 10.1186/s13578-021-00532-5 (PMC7869483; doi:10.1186/s13578-021-00532-5)
Supplement: Supplementary file 2 — Additional file 2: Figure S2. Pre-incubation of platelets with R11-S24 had no effect on thrombin-induced fibrinogen binding and platelet aggregation. Platelets were pre-incubated with R11, R11-S24 or R11-S24A peptide then stimulated with thrombin (0.05 U/ml). Fibrinogen binding was determined by flow cytometry. Platelet aggregation was recorded by a platelet aggregometer. [file 13578_2021_532_MOESM2_ESM.pdf]

Fig. S2

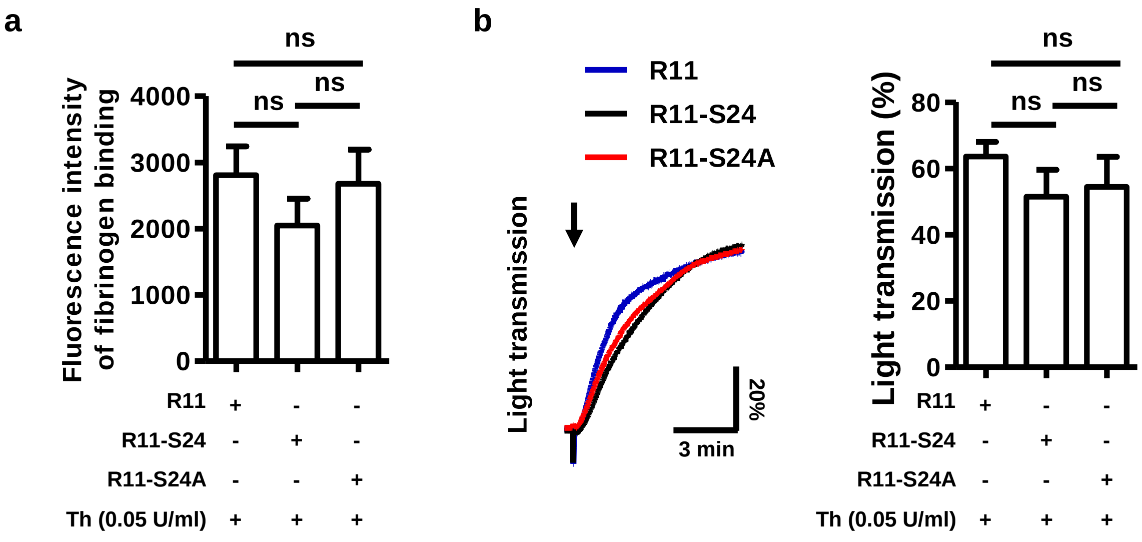

Fig. S2 Pre-incubation of platelets with R11-S24 had no effect on thrombin-induced fibrinogen binding and platelet aggregation. **a** Platelets were pre-incubated with R11, R11-S24 or R11-S24A peptide then stimulated with thrombin (0.05 U/ml) at 500 rpm for 10 min. Platelets were then diluted 10-fold by Tyrode's buffer and stained with Alexa Fluor™ 488 conjugated fibrinogen at RT for 20 min followed by flow cytometry. The data represent the mean ± SEM of 3 independent experiments. **b** Platelets were pre-incubated with R11, R11-S24 or R11-S24A peptide then stimulated with thrombin (0.05 U/ml) for 10 min. Arrows indicate the addition of thrombin. Platelet aggregation was recorded by a platelet aggregometer (Chrono-Log). The data represent the mean ± SEM of 4 independent experiments. ns, no significance
